# Outcomes of long-term nivolumab and subsequent chemotherapy in Japanese patients with head and neck cancer: 2-year follow-up from a multicenter real-world study

**DOI:** 10.1007/s10147-021-02047-y

**Published:** 2021-11-13

**Authors:** Ryuji Yasumatsu, Yasushi Shimizu, Nobuhiro Hanai, Shin Kariya, Tomoya Yokota, Takashi Fujii, Kiyoaki Tsukahara, Mizuo Ando, Kenji Hanyu, Tsutomu Ueda, Hitoshi Hirakawa, Shunji Takahashi, Takeharu Ono, Daisuke Sano, Moriyasu Yamauchi, Akihito Watanabe, Koichi Omori, Tomoko Yamazaki, Nobuya Monden, Naomi Kudo, Makoto Arai, Syuji Yonekura, Takahiro Asakage, Takahiro Nekado, Takayuki Yamada, Akihiro Homma

**Affiliations:** 1 Department of Otolaryngology, Graduate School of Medical Sciences, Kyushu University, Fukuoka, Japan; 2 Department of Medical Oncology, Faculty of Medicine and Graduate School of Medicine, Hokkaido University, Sapporo, Japan; 3 Department of Head and Neck Surgery, Aichi Cancer Center Hospital, Nagoya, Japan; 4 Department of Otolaryngology, Head and Neck Surgery, Okayama University Hospital, Okayama, Japan; 5 Division of Gastrointestinal Oncology, Shizuoka Cancer Center, Shizuoka, Japan; 6 Department of Head and Neck Surgery, Osaka International Cancer Institute, Osaka, Japan; 7 Department of Otorhinolaryngology, Head and Neck Surgery, Tokyo Medical University, Tokyo, Japan; 8 Otorhinolaryngology and Head and Neck Surgery, The University of Tokyo Hospital, Tokyo, Japan; 9 Head and Neck Oncology Center, International University of Health and Welfare, Mita Hospital, Tokyo, Japan; 10 Department of Otorhinolaryngology, Head and Neck Surgery, Hiroshima University Hospital, Hiroshima, Japan; 11 Department of Otorhinolaryngology, Head and Neck Surgery, University of the Ryukyus Hospital, Okinawa, Japan; 12 Department of Medical Oncology, Cancer Institute Hospital of the Japanese Foundation for Cancer Research, Tokyo, Japan; 13 Department of Otolaryngology, Head and Neck Surgery, Kurume University Hospital, Kurume, Japan; 14 Otolaryngology, Head and Neck Surgery, Yokohama City University Hospital, Yokohama, Japan; 15 Department of Otolaryngology, Head and Neck Surgery, Saga University Hospital, Saga, Japan; 16 Department of Otolaryngology, Head and Neck Surgery, Keiyukai Sapporo Hospital, Sapporo, Japan; 17 Department of Otolaryngology, Head and Neck Surgery, Kyoto University Hospital, Kyoto, Japan; 18 Division of Head and Neck Cancer Oncology, Miyagi Cancer Center, Sendai, Japan; 19 Department of Head and Neck Surgery, National Hospital Organization Shikoku Cancer Center, Matsuyama, Japan; 20 Department of Otorhinolaryngology, Hirosaki University Graduate School of Medicine, Hirosaki, Japan; 21 Department of Medical Oncology, Chiba University Hospital, Chiba, Japan; 22 Department of Otorhinolaryngology, Head and Neck Surgery, Chiba University Hospital, Chiba, Japan; 23 Department of Head and Neck Surgery, Tokyo Medical and Dental University Medical Hospital, Tokyo, Japan; 24 Medical Affairs, Ono Pharmaceutical Co., Ltd, Osaka, Japan; 25 Japan Medical and Development, Bristol-Myers Squibb K.K, Tokyo, Japan; 26 Department of Otolaryngology, Head and Neck Surgery, Faculty of Medicine and Graduate School of Medicine, Hokkaido University, Kita15 Nishi7, Kita-Ku, Sapporo, Hokkaido 060-8638 Japan; 27 Present Address: Department of Otolaryngology-Head and Neck Surgery, Okayama University Graduate School of Medicine, Okayama, Japan

**Keywords:** Nivolumab, Long-term survivors, Recurrent or metastatic head and neck cancer, Subsequent chemotherapy

## Abstract

**Background:**

We have previously reported the effectiveness and safety of nivolumab in patients with head and neck cancer (HNC) in real-world clinical practice in Japan. Here, we report long-term outcomes from this study in the overall population and subgroups stratified by subsequent chemotherapy.

**Methods:**

In this multicenter, retrospective observational study, Japanese patients with recurrent or metastatic (R/M) HNC receiving nivolumab were followed up for 2 years. Effectiveness endpoints included overall survival (OS), OS rate, progression-free survival (PFS), and PFS rate. Safety endpoints included the incidence of immune-related adverse events (irAEs).

**Results:**

Overall, 256 patients received a median of 6.0 doses (range: 1–52) of nivolumab over a median duration of 72.5 days (range: 1–736). Median OS was 9.5 months [95% confidence interval (CI) 8.2–12.0] and median PFS was 2.1 months (95% CI 1.8–2.7). A significant difference between 2-year survivors (*n* = 62) and non-2-year survivors was observed by median age (*P* = 0.0227) and ECOG PS (*P* = 0.0001). Of 95 patients who received subsequent chemotherapy, 54.7% received paclitaxel ± cetuximab. The median OS and PFS from the start of paclitaxel ± cetuximab were 6.9 months (95% CI 5.9–11.9) and 3.5 months (95% CI 2.3–5.5), respectively. IrAEs were reported in 17.2% of patients. Endocrine (7.0%) and lung (4.3%) disorders were the most common irAEs; kidney disorder (*n* = 1) was newly identified in this follow-up analysis.

**Conclusions:**

Results demonstrated the long-term effectiveness of nivolumab and potential effectiveness of subsequent chemotherapy in patients with R/M HNC in the real-world setting. Safety was consistent with that over the 1-year follow-up.

**Supplementary Information:**

The online version contains supplementary material available at 10.1007/s10147-021-02047-y.

## Introduction

Nivolumab is a fully human immunoglobulin G4 monoclonal antibody that targets programmed cell death protein-1. Nivolumab was approved in March 2017 in Japan for the treatment of unresectable recurrent or distant metastatic (R/M) head and neck cancer (HNC) that had progressed following chemotherapy. This approval was based on the survival benefits and manageable safety profile demonstrated in the global phase 3 CheckMate 141 study in patients with R/M squamous cell carcinoma of the head and neck (SCCHN) whose disease had progressed within 6 months after platinum-based chemotherapy [1]. The 2-year follow-up results of the CheckMate 141 study confirmed the long-term survival benefits of nivolumab [2]. In the CheckMate 141 study, long-term (2-year) follow-up data were reported for the overall population [2], as well as for the Asian subpopulation [3]. However, only 27 Japanese patients were enrolled in the study. Therefore, the long-term survival data of nivolumab in Japanese patients need to be investigated in a real-world setting.

In the 2-year follow-up analysis of CheckMate 141, the baseline characteristics between long-term survivors (LTSs), who were alive (in survival follow-up) at 2 years, and the overall population was investigated, but no difference was observed [2]. On the other hand, there are no reports related to the baseline characteristics between LTSs and non-LTSs in Japanese clinical practice. Therefore, prognostic factors for long-term survival in Japanese patients remain unknown. In Japan, nivolumab use is not limited to patients with squamous cell carcinoma (SCC); it is also used in patients with non-SCC, who were not included in CheckMate 141 [4]. The subgroup analysis results for SCC and non-SCC at the 1-year follow-up have been reported previously, whereby the effectiveness between patients with SCC and non-SCC was similar, with no statistically significant differences [5]. Considering the approved indication of nivolumab in Japan, effectiveness and prognostic factors according to SCC and non-SCC in long-term survivors need to be evaluated.

Recently, several studies have demonstrated the efficacy of subsequent chemotherapy in HNC following immunotherapy [6–9]. Saleh et al. reported an objective response rate (ORR) of 30% in patients who received chemotherapy after disease progression on immunotherapy, which was three to five times higher than that in those who had received chemotherapy as a second-line treatment in clinical trials [6]. Similar studies in Japan have demonstrated the efficacy of subsequent chemotherapy following immunotherapy in patients with HNC; however, these studies included only a small number of Japanese patients [7–9]. Furthermore, in patients with R/M HNC in whom disease progresses following first-line therapy, treatment options remain heterogeneous, and treatment patterns and outcomes show substantial variations across countries [10]. Therefore, the outcomes of subsequent chemotherapy following nivolumab treatment in the Japanese clinical setting is an important clinical question to be answered.

Although immune-related adverse events (irAEs) are frequently reported within a few weeks to months of initiating treatment with immune checkpoint inhibitors, delayed-onset irAEs sometimes occur after treatment discontinuation [11]. The onset of irAEs after nivolumab discontinuation has not been elucidated in patients with R/M HNC in a real-world clinical setting. Thus, effectiveness and safety data of nivolumab treatment over a long-term follow-up are warranted.

We recently reported the 1-year results from a real-world study in Japanese patients with HNC receiving nivolumab treatment to fill the data gap between clinical trials and real-world settings [5, 12]. Here, we report the long-term (2-year) effectiveness and safety results from this real-world study, including the effectiveness of subsequent chemotherapy.

## Patients and methods

### Study design

This was a multicenter, non-interventional, retrospective study conducted at 23 centers across Japan [5]. This study was conducted in accordance with the Ministerial Ordinance (number 171, issued on December 20, 2004) on Good Post-marketing Study Practice, Ministry of Health, Labour and Welfare, Japan, and the ethical principles of the Declaration of Helsinki. The study protocol was reviewed and approved by the institutional review board/independent ethics committee at each study site. Informed consent was not obtained; however, patients were given the opportunity to decline to have their clinical records used for research (opt-out consent provision). This study was registered at ClinicalTrials.gov (NCT03569436) and in the University hospital Medical Information Network Clinical Trials Registry (UMIN-CTR; UMIN000032600).

Patients with R/M HNC with disease progression on or after platinum-based therapy and who were treated with nivolumab at least once for the first time between Jul 1, 2017, and Dec 31, 2017, were included in the study, except those who had participated in a clinical trial assessing antineoplastic therapy [5]. Patient data from baseline until the most recent visit were collected in electronic case report forms from medical charts. The data cutoff date was 2 years after the first treatment with nivolumab in each patient.

### Assessments and endpoints

Primary endpoints were effectiveness and safety [5]. Effectiveness endpoints included best overall response (BOR), progression-free survival (PFS), and overall survival (OS) according to the investigator-assessed Response Evaluation Criteria in Solid Tumours (RECIST) 1.1 criteria [13]. Subgroup analyses were conducted to investigate the differences in baseline characteristics between 2-year survivors and non-2-year survivors and the effectiveness of subsequent chemotherapy. At the 2-year follow-up, patients who were alive were defined as 2-year survivor, while those who were not alive were defined as non-2-year survivors.

Safety endpoint included the incidence of irAEs. AEs were collected if they occurred up to 100 days after the last dose of nivolumab or up to the survey date, whichever was earlier. AEs were classified according to the International Council for Harmonisation of Technical Requirements for Pharmaceuticals for Human Use and Medical Dictionary for Regulatory Activities Japanese edition (MedDRA/J) version 21.0. The onset of irAEs was considered delayed in case of onset beyond 4 weeks of nivolumab discontinuation in patients who received subsequent systemic therapy other than nivolumab.

### Statistical analysis

Effectiveness and safety analyses were conducted in all patients who received ≥ 1 dose of nivolumab. Continuous variables were summarized as number, mean, and standard deviation; and categorical variables, as number and percentage. OS and PFS were estimated using the Kaplan–Meier method and expressed as the number and proportion of patients who survived to a specific point in time and median duration, with the corresponding two-sided 95% confidence interval (CI). 95% CI was calculated using the LOGLOG transformation. For the subgroup analysis to investigate differences between 2-year survivors and non-2-year survivors by baseline characteristics, *P* values were calculated using the Chi-square test and the Wilcoxon rank-sum test. Statistical analyses were conducted using SAS version 9.4 (SAS Institute, Tokyo, Japan).

## Results

### Patient characteristics

Overall, 256 patients from 23 clinical institutions were registered. Table 1 shows baseline demographics and patient characteristics. At the 2-year follow-up, 8.6% (*n* = 22) of enrolled patients were still receiving nivolumab treatment. Of the 234 patients (91.4%) who discontinued nivolumab treatment at 2 years, 179 (76.5%) had disease progression.

**Table 1:** Baseline characteristics stratified by survival status

Characteristic	All patients (*n* = 256)			
		Non-2-year survivors (*n* = 194)	2-year survivors (*n* = 62)	*P* value
Sex				0.7417
Male	202 (78.9)	154 (79.4)	48 (77.4)	
Female	54 (21.1)	40 (20.6)	14 (22.6)	
Age*, years, median (range)	66 (20–84)	65 (20–84)	67 (33–79)	0.0227
Age category				
< 65 years	114 (44.5)	94 (48.5)	20 (32.3)	
≥ 65 and < 75 years	118 (46.1)	79 (40.7)	39 (62.9)	
≥ 75 years	24 (9.4)	21 (10.8)	3 (4.8)	
ECOG PS*				0.0001
0	118 (46.1)	78 (40.2)	40 (64.5)	
1	97 (37.9)	77 (39.7)	20 (32.3)	
≥ 2	31 (12.1)	31 (16.0)	0 (0.0)	
Unknown	10 (3.9)	8 (4.1)	2 (3.2)	
Primary site				
Hypopharynx	64 (25.0)	50 (25.8)	14 (22.6)	0.6133
Oral cavity	56 (21.9)	44 (22.7)	12 (19.4)	0.5814
Oropharynx	40 (15.6)	30 (15.5)	10 (16.1)	0.9001
Salivary gland	23 (9.0)	17 (8.8)	6 (9.7)	0.8265
Larynx	21 (8.2)	17 (8.8)	4 (6.5)	0.5637
Maxillary sinus	14 (5.5)	11 (5.7)	3 (4.8)	0.8021
Nasopharynx	19 (7.4)	11 (5.7)	8 (12.9)	0.0586
Others	19 (7.4)	14 (7.2)	5 (8.1)	0.8245
Previous treatment				
Surgery	176 (68.8)	132 (68.0)	44 (71.0)	0.6652
Cetuximab	155 (60.5)	124 (63.9)	31 (50.0)	0.0510
Chemoradiation therapy	144 (56.3)	107 (55.2)	37 (59.7)	0.5320
Radiation therapy	94 (36.7)	74 (38.1)	20 (32.3)	0.4026

*Significant differences were observed between non-2-year survivors and 2-year survivors

Data are *n* (%) unless specified otherwise

*ECOG PS* Eastern Cooperative Oncology Group performance status

### Treatment exposure

Seventy patients (27.3%) received nivolumab as the first-line treatment for R/M HNC, 110 (43.0%) as second-line, and 76 (29.7%) as third- or later-line treatment [5]. Patients received a median of 6.0 doses (range: 1–52) of nivolumab over a median duration of 72.5 days (range: 1–736) of nivolumab treatment.

### Effectiveness of nivolumab in the overall population

**Fig. 1:**
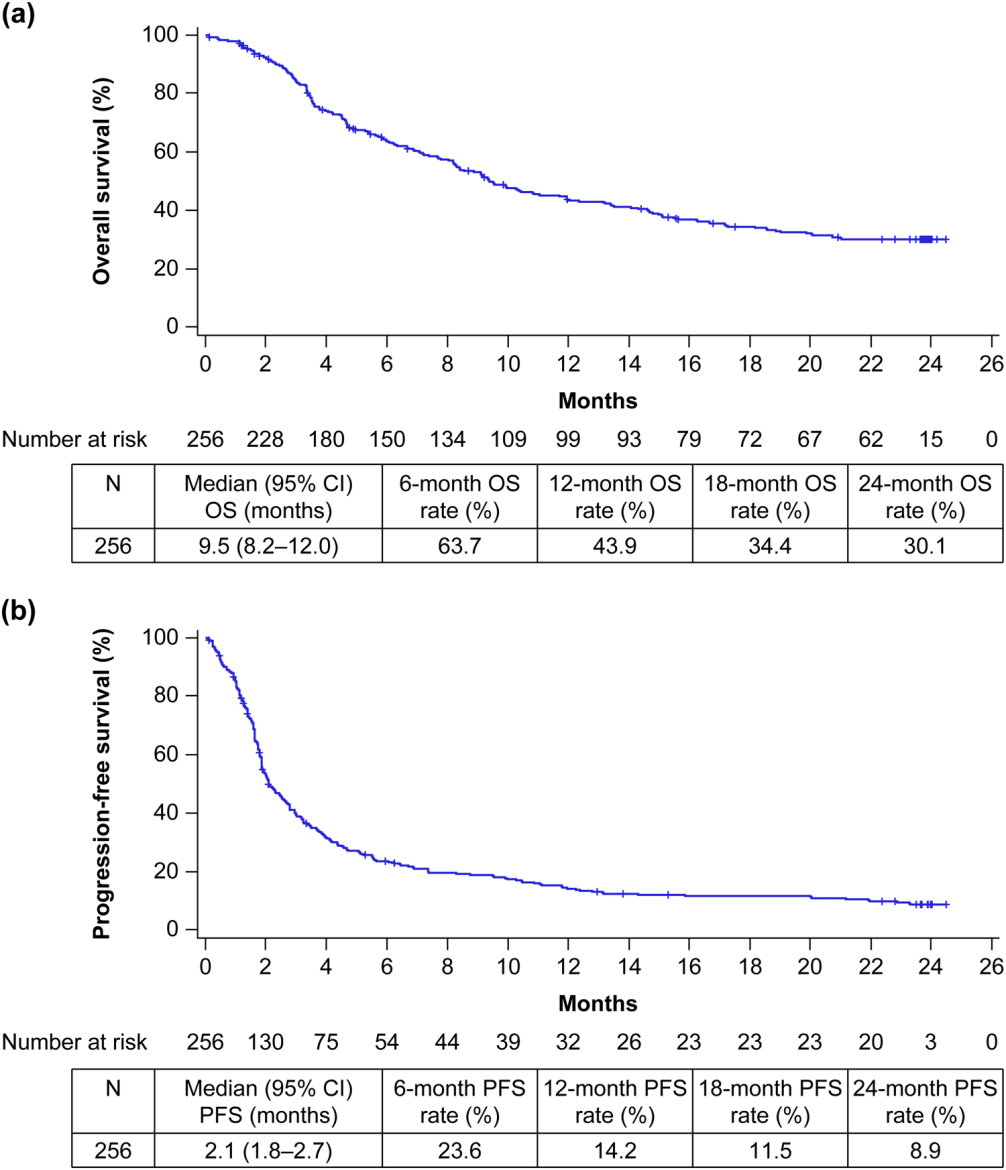
Kaplan–Meier curves in the overall population: **a **OS and **b** PFS. Survival curves were plotted based on the last survival confirmation date. Two-year survivors are shown as censored at 24 months. *CI* confidence interval, *OS* overall survival, *PFS* progression-free survival

Table 2 shows the effectiveness of nivolumab represented by the BOR, ORR, and disease control rate (DCR). The ORR was 16.1% (95% CI 11.6–21.6) and the DCR was 43.0% (95% CI 36.5–49.8; Table 2). The median OS was 9.5 months (95% CI 8.2–12.0) and the estimated 24-month OS rate was 30.1% (Fig. 1a). The median PFS was 2.1 months (95% CI 1.8–2.7) and the estimated 24-month PFS rate was 8.9% (Fig. 1b).

**Table 2:** Effectiveness of nivolumab treatment during the 2-year follow-up period

	Overall (*n* = 256)	2-year survivors (*n* = 62)
Evaluable patients	223 (87.1)	60 (23.4)
BOR		
Complete response	3 (1.3)	3 (5.0)
Partial response	33 (14.8)	25 (41.7)
Stable disease	60 (26.9)	21 (35.0)
Progressive disease	127 (57.0)	11 (18.3)
ORR	36 (16.1) 95% CI 11.6–21.6	28 (46.7) 95% CI 33.7–60.0
DCR	96 (43.0) 95% CI 36.5–49.8	49 (81.7) 95% CI 69.6–90.5

Data are *n* (%)

Response rates were calculated in evaluable patients

*BOR* best overall response, *CI* confidence interval, *DCR* disease control rate, *ORR* objective response rate

### Effectiveness by subgroups

Of the 256 patients, 62 were alive at the 2-year follow-up analysis. The BOR, ORR, and DCR in 60 patients of 2-year survivors are shown in Table 2. The BOR in 2-year survivors was complete response in three (5.0%), partial response in 25 (41.7%), and stable disease in 21 (35.0%) patients (Table 2). The ORR and DCR in 2-year survivors were 46.7% (95% CI 33.7–60.0) and 81.7% (95% CI 69.6–90.5), respectively (Table 2). Significant differences between 2-year survivors and non-2-year survivors were observed (Table 1) by age (*P* = 0.0227) and Eastern Cooperative Oncology Group performance status (ECOG PS; *P* = 0.0001). There were no patients with ECOG PS ≥ 2 in 2-year survivors, while 16% of patients had ECOG PS ≥ 2 in non-2-year survivors.

This study included 29 patients with non-SCC in addition to patients with SCC (*n* = 217). Effectiveness according to histological type (SCC vs. non-SCC) is shown in Online Resources 1–3. Effectiveness was similar between both groups, with no statistically significant differences; for instance, the median OS was 9.1 months (95% CI 7.4–10.4) in patients with SCC and 15.1 months [95% CI 4.5–not reached (NR)] in patients with non-SCC. A total of 50 patients with SCC were alive at the 2-year follow-up analysis. Significant differences between 2-year survivors and non-2-year survivors in SCC were observed by age (*P* = 0.0326), nasopharynx as primary site (*P* = 0.0388), and ECOG PS (*P* = 0.0007) (Online Resource 4).

### Effectiveness of subsequent chemotherapy

**Fig. 2:**
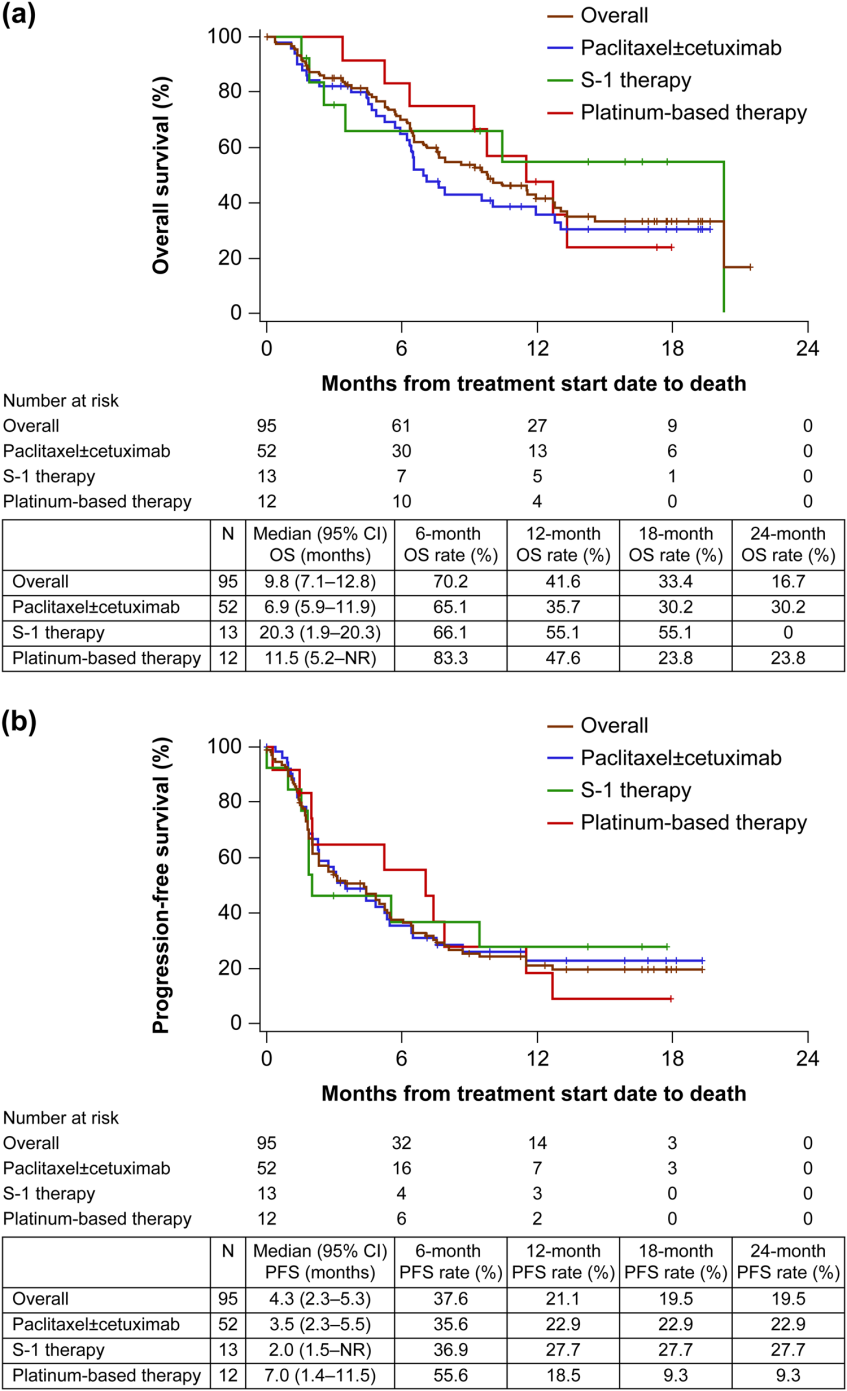
Kaplan–Meier curves in patients who received chemotherapy following nivolumab treatment: **a** OS and **b** PFS. *CI* confidence interval, *NR* not reached, *OS* overall survival, *PFS* progression-free survival

Ninety-five patients (37.1%) received chemotherapy following nivolumab treatment (Table 3). Of these, 54.7% (*n* = 52) received paclitaxel ± cetuximab as subsequent chemotherapy (8/52 patients received only paclitaxel; data not shown). Figure 2 shows the median OS and PFS from the start date of subsequent chemotherapy. In patients who received subsequent chemotherapy, the median OS was 9.8 months (95% CI 6.9–12.8; Fig. 2a) and the median PFS was 4.3 months (95% CI 2.3–5.3; Fig. 2b). In patients who received paclitaxel ± cetuximab as subsequent chemotherapy, the median OS was 6.9 months (95% CI 5.9–11.9) and the estimated 24-month OS rate was 30.2% (Fig. 2a); the median PFS was 3.5 months (95% CI 2.3–5.5) and the estimated 24-month PFS rate was 22.9% (Fig. 2b). In patients who received platinum-based therapy or S-1 therapy as subsequent chemotherapy, the median OS was 11.5 months (95% CI 5.2–NR) and 20.3 months (95% CI 1.9–20.3), respectively, with an estimated 24-month OS rate of 23.8% and 0%, respectively (Fig. 2a); the median PFS was 7.0 months (95% CI 1.4–11.5) and 2.0 months (95% CI 1.5–NR), respectively, with an estimated 24-month PFS rate of 9.3% and 27.7%, respectively (Fig. 2b).

**Table 3:** Effectiveness of subsequent chemotherapy

	Overall	Paclitaxel ± cetuximab	S-1 therapy	Platinum-based therapy
Total patients	95 (100.0)	52 (54.7)	13 (13.7)	12 (12.6)
Evaluable patients, *n* (%)	70 (73.7)	42 (44.2)	6 (6.3)	10 (10.5)
BOR				
Complete response	4 (5.7)	3 (7.1)	1 (16.7)	0 (0.0)
Partial response	19 (27.1)	12 (28.6)	1 (16.7)	4 (40.0)
Stable disease	17 (24.3)	13 (31.0)	0 (0.0)	2 (20.0)
Progressive disease	30 (42.9)	14 (33.3)	4 (66.7)	4 (40.0)
ORR	23 (32.9) 95% CI 22.1–45.1	15 (35.7) 95% CI 21.6–52.0	2 (33.3) 95% CI 4.3–77.7	4 (40.0) 95% CI 12.2–73.8
DCR	40 (57.1) 95% CI 44.7–68.9	28 (66.7) 95% CI 50.5–80.4	2 (33.3) 95% CI 4.3–77.7	6 (60.0) 95% CI 26.2–87.8

Data are *n* (%)

Response rates were calculated in evaluable patients

*BOR* best overall response, *CI* confidence interval, *DCR* disease control rate, *ORR* objective response rate

### Safety

**Fig. 3:**
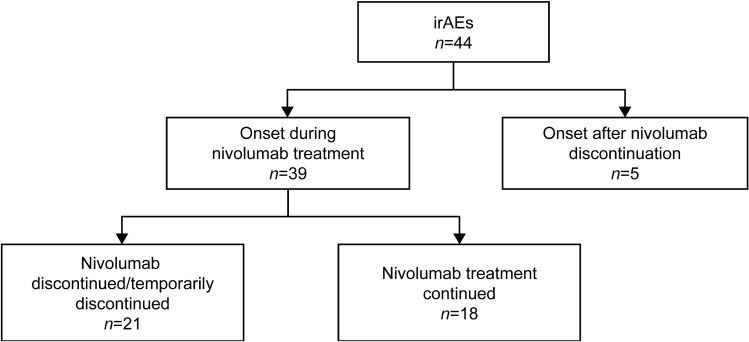
Distribution of irAEs in relation to onset and treatment status of nivolumab *irAE* immune-related adverse event

The overall incidence of any-grade irAEs was 17.2% (*n* = 44; Table 4). The most frequently reported irAEs were endocrine disorders in 18 (7.0%), lung disorders in 11 (4.3%), and liver and skin disorders in seven (2.7%) patients each. Kidney disorder (*n* = 1) was newly identified in this follow-up analysis. Grade ≥ 3 irAEs were reported in 18 (7.0%) patients (Table 4); lung disorders were the most common (2.3%) grade ≥ 3 irAE. Of 44 patients with irAEs of any grade, 39 reported irAEs during nivolumab treatment and five following nivolumab discontinuation (Fig. 3).

**Table 4:** Incidence of irAEs stratified by severity

irAE category (*n* = 256)	All grades	Grade ≥ 3
All patients with irAE	44 (17.2)	18 (7.0)
Lung disorder	11 (4.3)	6 (2.3)
Liver disorder	7 (2.7)	3 (1.2)
Skin disorder	7 (2.7)	2 (0.8)
Endocrine disorder	18 (7.0)	4 (1.6)
Neurological disorder	0 (0.0)	0 (0.0)
Kidney disorder	1 (0.4)	0 (0.0)
Gastrointestinal disorder	4 (1.6)	2 (0.8)
Blood disorder	2 (0.8)	1 (0.4)
Others	6 (2.3)	2 (0.8)

Data are *n* (%)

*irAE* immune-related adverse event

## Discussion

To our knowledge, this study provides the first long-term, real-world results of nivolumab in a large population with R/M HNC in Japan. The effectiveness of subsequent chemotherapies after nivolumab discontinuation as well as delayed irAEs were investigated to improve treatment management in patients with R/M HNC. In addition, patient characteristics that may impact long-term survival were also investigated.

The long-term real-world effectiveness of nivolumab in this study was comparable to that observed in the overall study population [2] and Asian subpopulation [3] in CheckMate 141 (2-year OS rate: 30.1, 16.9, 22.7%; median OS duration: 9.5, 7.7, 12.1 months; and ORR: 16.1, 13.3, 26.1%, respectively). The 2-year OS rate and ORR in the current study were higher than those observed in CheckMate 141. This difference could be due to the inclusion of patients with primary nasopharyngeal cancer [7.4% (*n* = 19)] in the current study. Patients with the nasopharynx as the primary site generally demonstrated a better OS rate and ORR than those with HNC at other primary sites in a subgroup analysis of the 1-year follow-up results from this study [5], but this patient population was not included in CheckMate 141. Thus, the difference in OS rate and ORR for the overall population between the current study and CheckMate 141 could be attributed to the inclusion and exclusion of patients with primary nasopharyngeal cancer. However, the efficacy of nivolumab in nasopharyngeal cancer should be elucidated in other confirmatory clinical trials.

In CheckMate 141 and the current study, 15.4 and 24.2%, respectively, of enrolled patients who received nivolumab were LTSs [2]. There was no observed difference in the baseline characteristics of the overall population and LTSs in the CheckMate 141 study [2]. In the current study, significant differences between non-2-year survivors and 2-year survivors in median age (*P* = 0.0227) and ECOG PS (*P* = 0.0001) were observed. A previous meta-analysis that investigated randomized clinical trials involving patients with cancer of several types reported that older patients (≥ 65 years) could benefit more from immunotherapy than younger patients (< 65 years) [14]. In the current analysis, a significant difference in age at baseline was observed between 2-year survivors and non-2-year survivors; however, the difference was only 2 years between the median age of 2-year survivors (67 years; range: 33–79) and that of non-2-year survivors (65 years; range: 20–84). Thus, it is unclear if this small but significant difference in age could be clinically meaningful.

In the current study, a significant difference was also observed in the baseline ECOG PS between 2-year survivors and non-2-year survivors. The previous 1-year follow-up analysis of this study showed that the ORR, PFS, and OS were numerically greater in patients with ECOG PS 0 than those in patients with ECOG PS 1 or ≥ 2 [5]. Similarly, a difference by ECOG PS in the efficacy of immunotherapy has been reported in patients with malignant pleural mesothelioma [15], melanoma [16], and non-small-cell lung cancer [17] as well as for those with SCCHN [7]. Fushimi et al. investigated prognostic factors for patients with R/M SCCHN who received salvage chemotherapy after nivolumab treatment and reported that patients with ECOG PS 0 showed better OS compared with those with ECOG PS 1 [7]. Patients with a good ECOG PS can safely receive subsequent chemotherapy following immunotherapy compared with those with a poor ECOG PS. Moreover, since the 30-day mortality is known to increase in patients on palliative chemotherapy with a poor ECOG PS [18], the efficacy of subsequent chemotherapy could likely be influenced by ECOG PS. Further studies are warranted to confirm this hypothesis. Subgroup analyses by histological type confirmed that effectiveness was similar between patients with SCC and non-SCC, with no statistically significant differences. Age and ECOG PS remained significant prognostic factors in patients with SCC as well as in the overall population.

Several recent studies have demonstrated the efficacy of subsequent chemotherapy following immunotherapy in patients with HNC [6–9]. A previous study reported a significantly longer OS (7.8 months, *P* = 0.0028, *n* = 25) in Japanese R/M SCCHN patients who received salvage chemotherapy following nivolumab treatment than that in patients who received best supportive care (3.5 months, *n* = 31) [7]. In the current study, paclitaxel ± cetuximab was the most frequently administered (*n* = 52) subsequent chemotherapy in patients who received chemotherapy following nivolumab. Median OS, median PFS, and ORR were 6.9 months, 3.5 months, and 35.7%, respectively, in patients who received paclitaxel ± cetuximab as subsequent chemotherapy. In a previous clinical study investigating the efficacy of a combination of paclitaxel and cetuximab as first-line treatment in patients with R/M SCCHN, median OS, median PFS, and ORR were 8.1 months, 4.2 months, and 54%, respectively [19]. In general, a late-line palliative chemotherapy results in poor prognosis [20]. In the present study, despite 72.7% of patients receiving nivolumab as second- or later-line treatment [5], paclitaxel ± cetuximab as subsequent chemotherapy demonstrated favorable effectiveness, which was consistent with the findings of a previous study that investigated a combination of paclitaxel and cetuximab as first-line treatment [19]. Several small retrospective studies in Japanese patients with R/M SCCHN have also reported the effectiveness of chemotherapy following treatment with immune checkpoint inhibitors [7–9]. Subsequent chemotherapy with or without cetuximab following treatment with immune checkpoint inhibitors, including nivolumab, demonstrated improved clinical responses, with overall response rates of 40.9% [8], 60.0% [9], and 36% [7], median OS of 7.3 months [7] and 14.5 months [8], and median PFS ranging from 2.3 months to 5.4 months [7–9]. Although a small number of patients in the current study also received subsequent chemotherapy with S-1 or platinum-based therapy (*n* = 13 and 12, respectively), the median OS and PFS were comparable with that achieved with paclitaxel ± cetuximab. Taken together with the findings from previous studies, these results support the higher chemosensitivity for subsequent therapies after immunotherapy. The higher effectiveness of chemotherapy following nivolumab treatment could be considered as one of the reasons for improved OS, as suggested by earlier studies [6, 21].

In the 1-year follow-up analysis, no new safety signals had been identified compared with CheckMate 141, with a median time to onset of irAEs of 8.7 weeks [5]. The irAE profile in the current study was largely consistent with that of the 1-year analysis. Kidney disorder was newly identified as an irAE and was reported in one patient. Late-onset irAEs are rare but have been reported following immunotherapy [22, 23]. The delayed onset of irAEs could be associated with prolonged receptor occupancy irrespective of undetectable serum levels of the immune checkpoint inhibitor, which may cause persistent immune activation even after treatment cessation [24]. In this study, five patients reported irAEs after nivolumab discontinuation, reinforcing the importance of careful monitoring for irAEs in patients even after discontinuation of nivolumab treatment.

Retrospective observational studies have several limitations, including the absence of a control group. Furthermore, our data were based on the assessments by individual physicians during their routine clinical practice, which may not always be complete or provide information for comparison and may contain measurement errors. Moreover, we preferentially included study centers that had a high number of patients with R/M HNC to allow for recruitment of an optimal number of patients, which may have unintentionally introduced a selection bias in the study.

## Conclusions

These 2-year follow-up results in Japanese patients with R/M HNC demonstrated that the real-world effectiveness and safety of nivolumab was consistent with that observed in CheckMate 141. Chemotherapy following nivolumab treatment may be effective in patients with R/M HNC.

## Supplementary Information

Below is the link to the electronic supplementary material.

Supplementary file1 (PDF 136 KB)

Supplementary file2 (PDF 178 KB)

Supplementary file3 (PDF 177 KB)

Supplementary file4 (PDF 170 KB)
