# Supplementary material for: Outcomes of long-term nivolumab and subsequent chemotherapy in Japanese patients with head and neck cancer: 2-year follow-up from a multicenter real-world study
Source: Int J Clin Oncol. 2021 Nov 13;27(1):95–104. doi: 10.1007/s10147-021-02047-y (PMC8732924; doi:10.1007/s10147-021-02047-y)
Supplement: Supplementary file 1 — Supplementary file1 (PDF 136 KB) [file 10147_2021_2047_MOESM1_ESM.pdf]

Online Resource 1

Outcomes of long-term nivolumab and subsequent chemotherapy in Japanese patients with head and neck cancer: 2-year follow-up from a multicenter real-world study

International Journal of Clinical Oncology

Ryuji Yasumatsu, Yasushi Shimizu, Nobuhiro Hanai, Shin Kariya, Tomoya Yokota, Takashi Fujii, Kiyoaki Tsukahara, Mizuo Ando, Kenji Hanyu, Tsutomu Ueda, Hitoshi Hirakawa, Shunji Takahashi, Takeharu Ono, Daisuke Sano, Moriyasu Yamauchi, Akihito Watanabe, Koichi Omori, Tomoko Yamazaki, Nobuya Monden, Naomi Kudo, Makoto Arai, Syuji Yonekura, Takahiro Asakage, Takahiro Nekado, Takayuki Yamada, Akihiro Homma

Corresponding Author: Akihiro Homma, E-mail: ak-homma@med.hokudai.ac.jp

Overall response rate among patients according to histology status

Histology	*N*	ORR (95% CI), %	BOR, *n* (%)			
			CR	PR	SD	PD
Non-SCC	29	10.7 (2.3–28.2)	1 (3.6)	2 (7.1)	11 (39.3)	14 (50.0)
SCC	217	16.1 (11.2–22.2)	2 (1.1)	28 (15.1)	46 (24.7)	110 (59.1)

BOR best overall response, *CI* confidence interval, *CR* complete response, *ORR* objective response rate, *PD* progressive disease, *PR* partial response, *SCC* squamous cell carcinoma, *SD* stable disease
