# Supplementary material for: Outcomes of long-term nivolumab and subsequent chemotherapy in Japanese patients with head and neck cancer: 2-year follow-up from a multicenter real-world study
Source: Int J Clin Oncol. 2021 Nov 13;27(1):95–104. doi: 10.1007/s10147-021-02047-y (PMC8732924; doi:10.1007/s10147-021-02047-y)
Supplement: Supplementary file 2 — Supplementary file2 (PDF 178 KB) [file 10147_2021_2047_MOESM2_ESM.pdf]

# Online Resource 2

**Outcomes of long-term nivolumab and subsequent chemotherapy in Japanese patients with head and neck cancer: 2-year follow-up from a multicenter real-world study**

*International Journal of Clinical Oncology*

Ryuji Yasumatsu, Yasushi Shimizu, Nobuhiro Hanai, Shin Kariya, Tomoya Yokota, Takashi Fujii, Kiyoaki Tsukahara, Mizuo Ando, Kenji Hanyu, Tsutomu Ueda, Hitoshi Hirakawa, Shunji Takahashi, Takeharu Ono, Daisuke Sano, Moriyasu Yamauchi, Akihito Watanabe, Koichi Omori, Tomoko Yamazaki, Nobuya Monden, Naomi Kudo, Makoto Arai, Syuji Yonekura, Takahiro Asakage, Takahiro Nekado, Takayuki Yamada, Akihiro Homma

**Corresponding Author:** Akihiro Homma, E-mail: ak-homma@med.hokudai.ac.jp

**Progression-free survival among patients according to histology status**

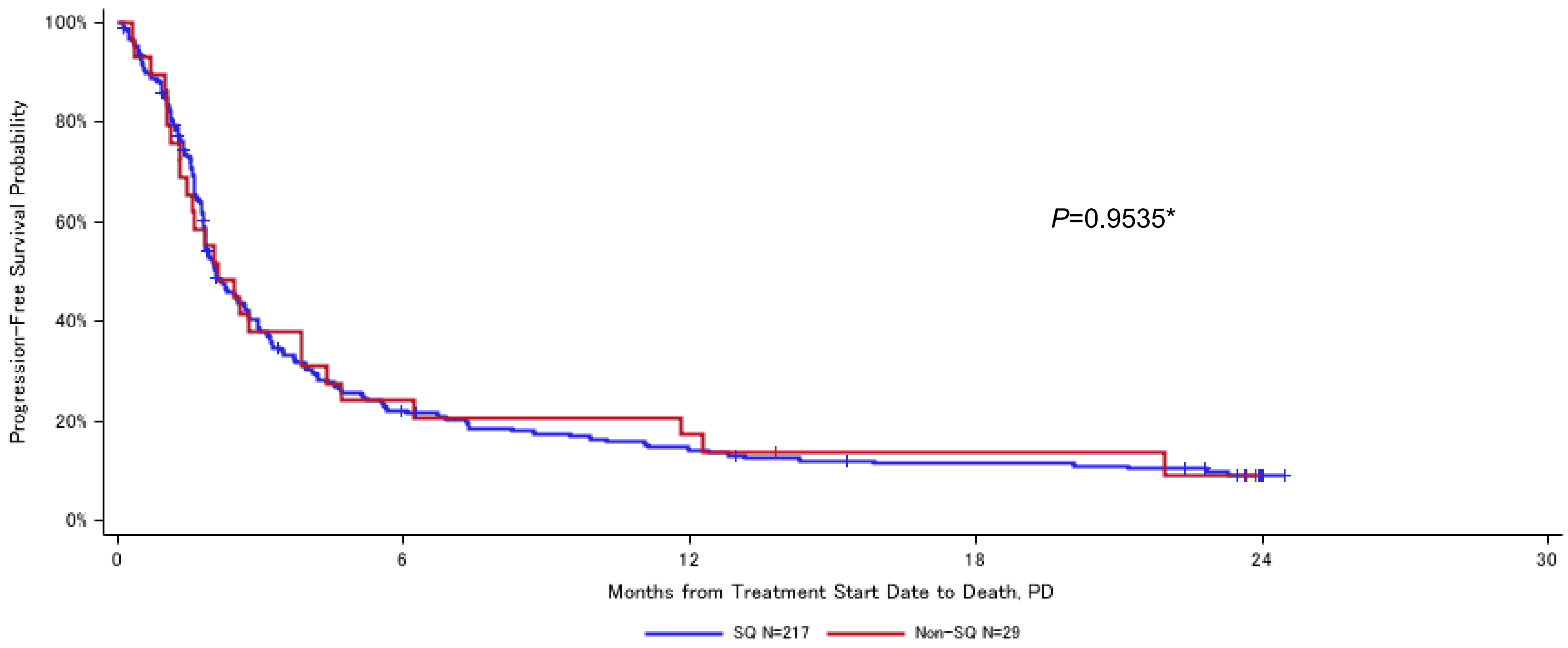


| Histology | *N* | Median PFS (95% CI), (months) | 6-month PFS rate (%) | 12-month PFS rate (%) | 18-month PFS rate (%) | 24-month PFS rate (%) |
|---|---|---|---|---|---|---|
| Non-SCC | 29 | 2.1 (1.3–3.8) | 24.1 | 17.2 | 13.8 | 9.2 |
| SCC | 217 | 2.1 (1.8–2.6) | 22.2 | 14.3 | 11.5 | 9.1 |

*PFS* progression-free survival, *SCC* squamous cell carcinoma, *log-rank test
