# Supplementary material for: Outcomes of long-term nivolumab and subsequent chemotherapy in Japanese patients with head and neck cancer: 2-year follow-up from a multicenter real-world study
Source: Int J Clin Oncol. 2021 Nov 13;27(1):95–104. doi: 10.1007/s10147-021-02047-y (PMC8732924; doi:10.1007/s10147-021-02047-y)
Supplement: Supplementary file 4 — Supplementary file4 (PDF 170 KB) [file 10147_2021_2047_MOESM4_ESM.pdf]

Online Resource 4

Outcomes of long-term nivolumab and subsequent chemotherapy in Japanese patients with head and neck cancer: 2-year follow-up from a multicenter real-world study

International Journal of Clinical Oncology

Ryuji Yasumatsu, Yasushi Shimizu, Nobuhiro Hanai, Shin Kariya, Tomoya Yokota, Takashi Fujii, Kiyoaki Tsukahara, Mizuo Ando, Kenji Hanyu, Tsutomu Ueda, Hitoshi Hirakawa, Shunji Takahashi, Takeharu Ono, Daisuke Sano, Moriyasu Yamauchi, Akihito Watanabe, Koichi Omori, Tomoko Yamazaki, Nobuya Monden, Naomi Kudo, Makoto Arai, Syuji Yonekura, Takahiro Asakage, Takahiro Nekado, Takayuki Yamada, Akihiro Homma

Corresponding Author: Akihiro Homma, E-mail: ak-homma@med.hokudai.ac.jp

Baseline characteristics in patients with SCC

Characteristic	Patients with SCC (*n*=217)			
		Non-2-year survivors (*n*=167)	2-year survivors (*n*=50)	*P* value
**Sex**				0.5863
Male	172 (79.3)	131 (78.4)	41 (82.0)	
Female	45 (20.7)	36 (21.6)	9 (18.0)	
**Age*, years, median (range)**	67 (24–84)	66 (24–84)	67 (44–78)	0.0326
**Age category**				
<65 years	87 (40.1)	73 (43.7)	14 (28.0)	
≥65 and <75 years	108 (49.8)	74 (44.3)	34 (68.0)	
≥75 years	22 (10.1)	20 (12.0)	2 (4.0)	
**ECOG PS***				0.0007
0	91 (41.9)	62 (37.1)	29 (58.0)	
1	91 (41.9)	72 (43.1)	19 (38.0)	
≥2	29 (13.4)	29 (17.4)	0 (0.0)	
Unknown	6 (2.8)	4 (2.4)	2 (4.0)	
**Primary site**				
Hypopharynx	59 (27.2)	47 (28.1)	12 (24.0)	0.5635
Oral cavity	54 (24.9)	43 (25.7)	11 (22.0)	0.5907
Oropharynx	35 (16.1)	26 (15.6)	9 (18.0)	0.6818
Salivary gland	5 (2.3)	4 (2.4)	1 (2.0)	0.8702
Larynx	21 (9.7)	17 (10.2)	4 (8.0)	0.6474
Maxillary sinus	11 (5.1)	9 (5.4)	2 (4.0)	0.6944
Nasopharynx*	19 (8.8)	11 (6.6)	8 (16.0)	0.0388
Others	13 (6.0)	10 (6.0)	3 (6.0)	0.9975

*Significant differences observed between non-2-year survivors and 2-year survivors in patients with SCC

Data are *n* (%) unless specified otherwise

SCC squamous cell carcinoma; *ECOG PS* Eastern Cooperative Oncology Group performance status
